# The ship domain in navigational safety assessment

**DOI:** 10.1371/journal.pone.0265681

**Published:** 2022-04-26

**Authors:** Miroslaw Wielgosz, Zbigniew Pietrzykowski

**Affiliations:** 1 Faculty of Navigation, Maritime University of Szczecin, Szczecin, Poland; 2 Faculty of Computer Science and Telecommunications, Maritime University of Szczecin, Szczecin, Poland; Second Institute of Oceanography Ministry of Natural Resources, CHINA

## Abstract

Decision support and decision making systems find increasingly more applications in various modes of transport. In this connection, criteria of movement safety assessment must be established for manned, unmanned, and autonomous vehicles, bearing in mind the specifics of each transport mode. Distance is one of the basic criteria. In sea transport these include the closest point of approach and ship’s domain understood as an area that should remain free of other objects. The authors examine the relationship between the declared domain and the actually maintained effective domain in a restricted area. They define the coefficient describing this relationship for different ship’s relative bearings in the form of a mathematical function. The formulated relationship allows determining an effective passing distance based on the declared, i.e., assumed passing distance. The determined relationship also enables identification of the declarative domain based on the effective one. These relationships may be used in decision support systems of manned ships, in remote ship control centres and decision-making systems of autonomous ships for the assessment of ship movement safety, planning collision avoidance manoeuvres and generation of safe trajectories.

## Introduction

Safe navigation requires continuous analysis and assessment of the situation by using various navigational aids available on board. Navigational situation analyses and assessment must be based on specific criteria. In restricted areas, the parameters commonly used in the process of situation analysis and assessment are insufficient. Their use in restricted waters is usually very difficult and requires a lot of experience in interpretation and practical application. Replacement of these parameters by an elliptical domain of the ship with the possibility of defining its basic parameters would significantly improve the process of assessing navigational safety. Electronic Chart Display and Information Systems (ECDIS) and simpler Electronic Chart Systems (ECS) offer the capabilities of defining and introducing new parameters that are complementary to or replace those currently used. The ship domain seems to attract more interest as a tool for analyzing and assessing navigational situations. The concept of declarative domain was introduced in [1], but the previously formulated definitions of domain indicate their declarative nature [2, 3]. According to [3], the ship’s domain is an area or space that navigators declare that they would like to maintain clear of other objects that affect or that may affect navigational safety. The effective domain is an area or space that navigators maintain clear of other objects. Looking at the concepts of declarative domain and effective domain, we can assume that the latter is the result of the fact that the navigator, apart from the declarative domain, adopts additional criteria and restrictions.

Knowledge of the relationships between ship domains identified in tests will allow one to determine the distance at which the ships will actually pass each other on any relative bearing, based on the passing distance assumed and desired by the navigator. This will lead to the creation of an effective domain model based on the declarative domain.

## Background

The essential factors affecting the shape and size of ship domain include ship size and speed and the type and parameters of the water area. The ship domain, as a criterion of navigational safety assessment, is of particular importance in areas of high vessel traffic intensity, where the ship has limited manoeuvring possibilities due to the occurrence of physical and conventional restrictions.

In the currently used and newly introduced devices and systems, the ship domain can be practically used for:

- current analysis and assessment of the situation;
- planning of anti-collision manoeuvres;
- use in navigational decision support systems.

There are several methods of determining the ship domain. These are: real tests, simulation tests, expert questionnaire surveys, analytical methods, and artificial intelligence methods. The article presents the results of simulation tests conducted for the development of the effective domain model and expert-based survey for the determination of the declarative domain. The results were summarized by formulating a universal relationship between the declarative domain and the effective domain of the ship.

### Literature review

The concept of ship domain appeared in the literature in the early 1970s. In 1971 Fujii and Tanaka published the results of research on the capacity of fairways [2]. Those authors observed that the so called effective domain affects traffic capacity. They defined the effective domain as "an area around a ship underway that most of the navigators preferred not to violate". Several factors and their impact on the shape and size of the domain were identified and described, including the size and the speed of the ship and hydrometeorological conditions.

The first scientific publication entirely dedicated to the ship domain was published in 1975 [3]. She defined the ship domain as an effective area around a ship that the navigator would like to maintain clear of other ships and fixed objects. The domain in restricted areas was examined by Coldwell analysing the impact of ship size on domain shape [4]. Research in real domains was initially conducted only in restricted areas, mainly due to the operational range of radar equipment.

Ship domains were determined based on observations and recordings of data from radars or Automatic Radar Plotting Aid (ARPA) devices installed on ships, land-based Vessel Traffic Systems (VTS) or research centres. The common development of electronic position determination systems and satellite global positioning systems in the early 1990s resulted in data recording and ship domain determination using fixed positions of ships. Such domains are referred to as effective domains. The wide access to Automatic Identification System (AIS) data enabled the tracking of real trajectories of ships, analysis of the domains, and the determination of safety levels. Hansen et al. presented the results of research using recorded AIS data in the form of density charts, where the length of the ship was used as a relative unit of distance [5]. Similar research was conducted by Silveira et al. [6] and Pietrzykowski et al. [7]. Simulation tests were presented in [8, 9] to determine safety levels and assess risks. The impact of ship size and speed on the shape and size of the domain was presented in [10]. At the same time research of declarative nature was conducted: questionnaires filled out by expert navigators. The ship domain as a criterion of navigational safety in an open sea area was described in [11]. The concept of hydrographic domains was introduced by Wielgosz [12], who also analysed the impact of the size of the area on ship domain [13].

In 1993 Zhao, Wu, and Wang [14] presented the theory of the fuzzy boundary of the domain, analysing factors affecting its shape and size. The definition of the fuzzy domain presented by Pietrzykowski broadened the concept of the fuzzy boundary of the domain [15]. This author also proposed the use of artificial neural networks with fuzzy logic. The concept of a dynamic fuzzy domain was introduced in [16].

Domains determined by analytical methods constitute a separate group. Liu et al. [17] presented a dynamic model of the ship domain for an analysis of vessel traffic in restricted areas. The expanded analytical models were presented by Wang et al. [18]. Wang also proposed the concept of dynamic quaternion domain [19, 20]. Zhang & Meng [21] presented a model of probabilistic ship domain. The use of the domain and artificial potential fields in anti-collision manoeuvres were presented by Wang et al. [22]. The various advancements in ship domains were reviewed in Szlapczynski & Szlapczynska [23] and Hörteborn et al. [24]. To date, nothing has been published on the relation/s between the declarative and effective domains of the ship.

Today, many research centers around the world are working on autonomous ships [25, 26]. Progress is possible due to the rapid development of modern technologies, including IT. Autonomous ships have already been tested and are put into service in local shipping operations. By International Maritime Organization (IMO) categories, the autonomy of these ships is distinguished by degree one, two and three. Autonomy mainly refers to navigational processes. In the module of autonomous navigation, key functionalities include the analysis and assessment of a navigational situation, the determination of safe ship movement trajectory, steering along a set trajectory, monitoring of the current navigational situation, and its prediction. For this purpose, it is necessary to establish criteria, in particular the criteria of navigational safety. This applies to the own ship and the other ship. With respect to other ships, safety criteria allow one to predict the behaviour of another ship and prevent the occurrence of a dangerous collision situation.

## Materials and methods

### The water area, types of ships, test station

**Fig 1:**
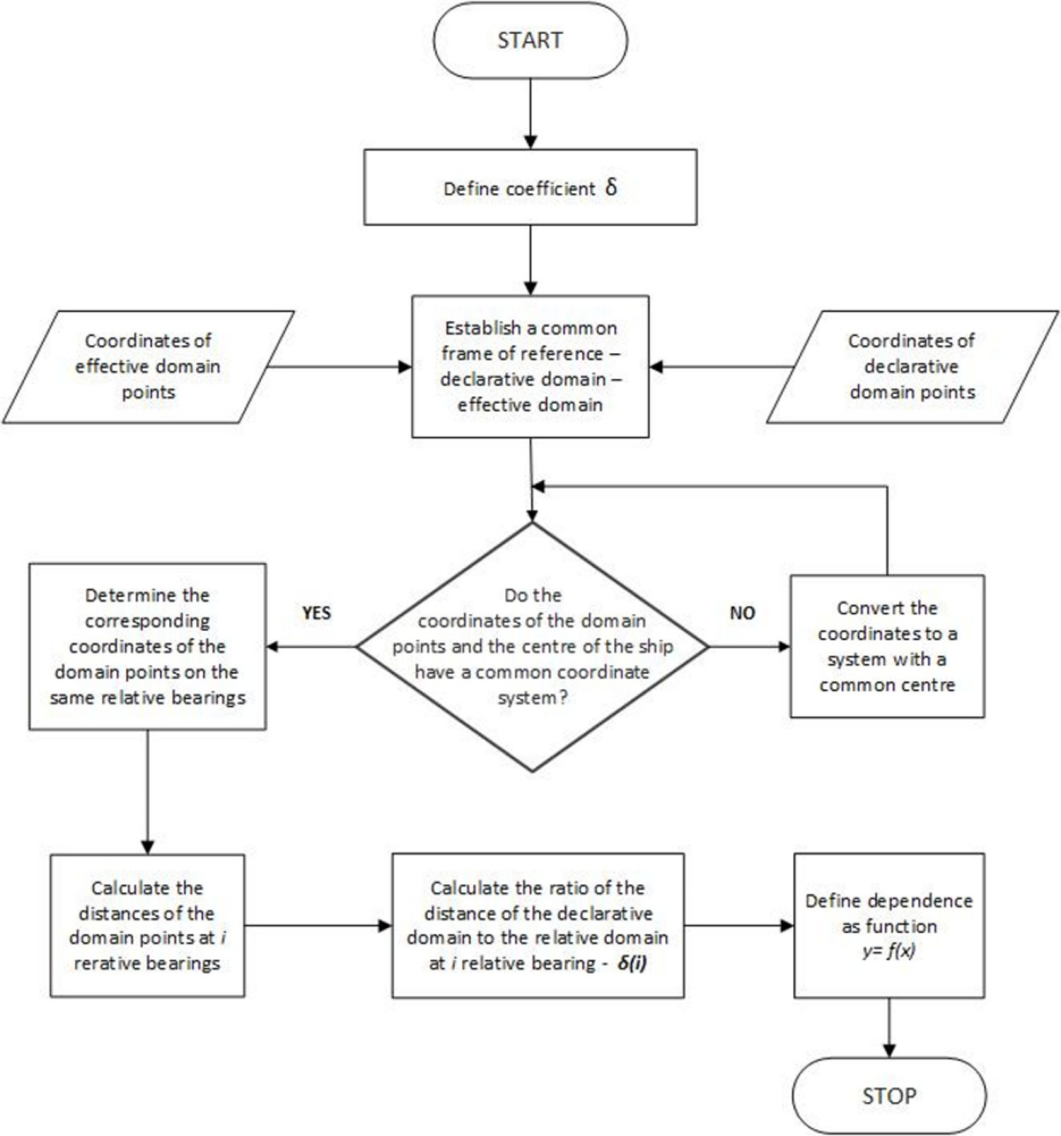
Absolute declarative domains of a medium ship.

**Fig 2:**
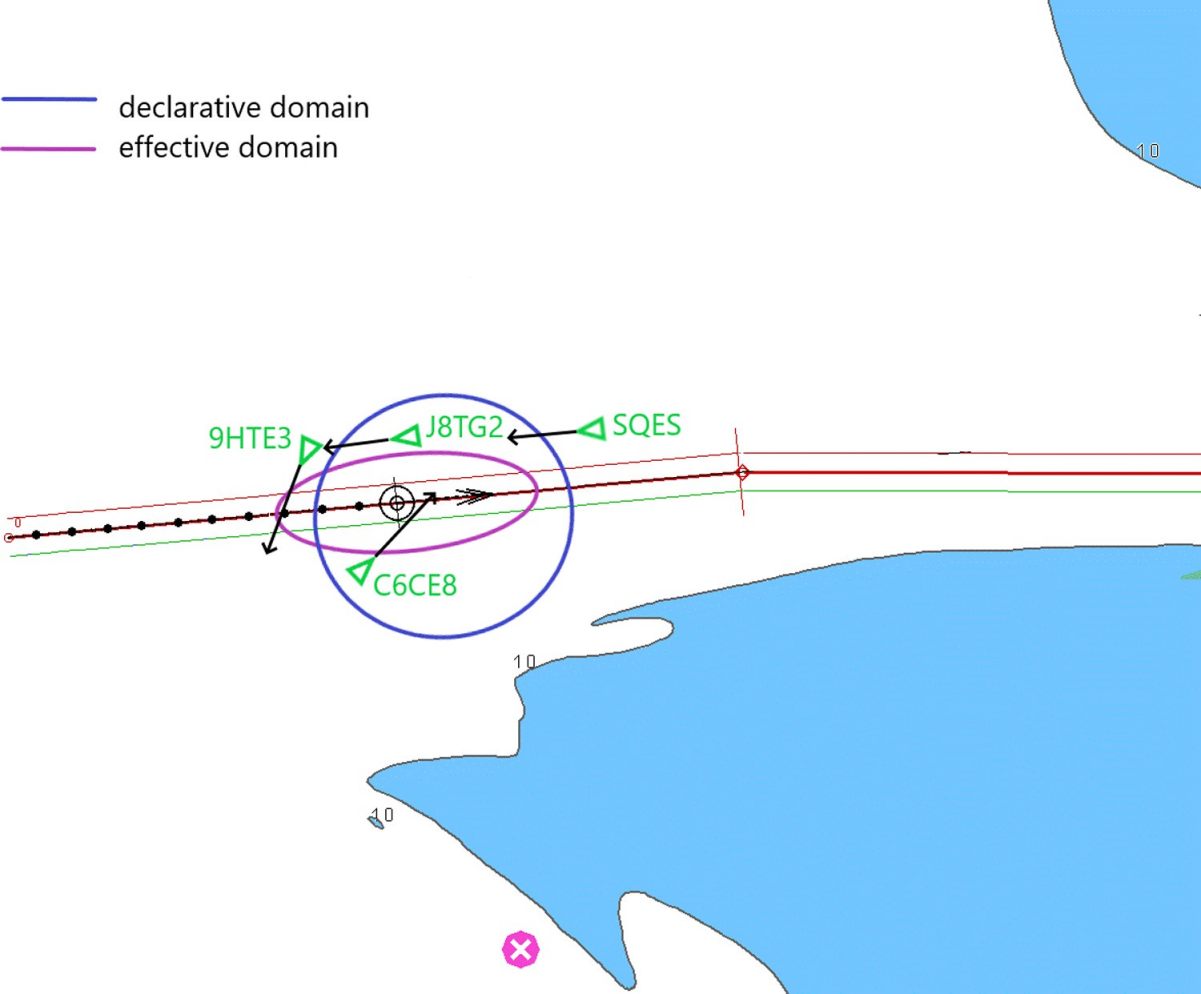
Relative declarative domains of a medium ship.

Two restricted areas were chosen for the questionnaires and simulation tests: the Singapore Strait and the Dover Strait. Both shipping areas, in terms of size, traffic intensity, and the presence of traffic separation schemes, are similar, and most navigators are familiar with them from experience. They are known for the highest vessel traffic intensity in the world. Creating the models of declarative and effective domains, the authors examined the impact of ship size and speed on the shape and size of the domain. In all the tests, the minimum, mean and maximum domains were determined. Our analysis included absolute domains (Fig 1) and relative domains (Fig 2), where the recorded distances were divided by ship length.

In both types of research three models of ships of different sizes were used, further referred to as large, medium and small ships (Table 1).

**Table 1:** Technical and operational parameters of the ships used in the tests.

	Ship size		
Parameter	large	medium	Small
Length [m]	261.3	173.5	95.0
Breadth [m]	48.0	23.0	13.0
Draught [m]	9.0	8.1	3.7
Speed [m/s]	8.2	9.5	5.6

To examine the impact of ship size, three groups of scenarios and questionnaires were prepared:

- large ship encounters–a large ship with a large ship;
- medium ship encounters–involving two medium size ships;
- small ship encounters–a small ship meets another small ship.

To examine the impact of ship speed variants, three separate groups of scenarios and questionnaires were prepared (six encounters in each group):

- low-high speed–manoeuvring ship proceeds at a speed approximately twice slower than the non-manoeuvring ship steaming “full ahead”;
- equal speeds–both ships move initially at the same speed;
- high-low speed–manoeuvring ship proceeds at ‘manoeuvring full ahead’, approximately twice faster than that of the non-manoeuvring ship’s speed.

### Declarative domain

To determine declarative domains, the questionnaire-based survey was conducted among participants in training courses at the Marine Officer Training Centre, the Maritime University of Szczecin, currently employed in shipping companies as operational or management level deck personnel. The questionnaires used to collect data for the article were voluntary and conducted anonymously among navigators improving their professional qualifications during supplementary training courses. The questions referred to the passing distance of ships on selected heading angles that the navigators wished to maintain in a collision avoiding manoeuvre. The research was conducted for different encounter situations. Eight characteristic, easy to identify relative bearings were selected: 000°, 045°, 090°, 135°, 180°, 225°, 270°, 315°. A total of 153 questionnaires were collected, each containing three sets of declared distances at a/m relative bearings for different ship size and speed relations. Finally, 459 individual ship domains for three ship sizes and different speed relations were collected. Calculations were carried out in nautical miles converted to meters, and using the relative distance units (a multiple of ship length), where distances found in a questionnaire form were divided by the respective ship length. The questionnaires are included in the S1–S3 Appendices. The method of determining declarative domains is presented in [1].

The obtained sets of points were placed on collective charts in the so-called radar system, presenting distances as the function of relative bearings for all the tested angles (Figs 1 and 2, dashed lines). Both the charts and the questionnaires were made in the head-up display, transparent and unambiguous for navigators. Linear approximation was applied to obtain various domains: maximum, mean and minimum, for each of the tested size of ship and speed variant. To get the results corresponding to simulation tests, declarative domains of various size ships were investigated in two categories: absolute, relative. The example given herein involves a medium ship. The parameters of ellipses obtained by approximation of absolute and relative domains of a medium ship are given in Table 2.

**Table 2:** Parameters of medium ship absolute and relative declarative domains after approximation.

Absolute domains		*x*_*0*_	*y*_*0*_	*a*	*b*
		[m]	[m]	[m]	[m]
	mean	358	95	1172	1086
	max	517	206	1812	1687
	min	252	0	6741	598
Relative domains		*x_0_*	*y_0_*	*a*	*b*
		[L]	[L]	[L]	[L]
	mean	2.1	0.6	6.8	6.3
	max	3.0	1.2	10.4	9.7
	min	1.5	0.0	3.9	3.5

L–length of the ship.

Based on the data presented in Table 3, elliptical absolute and relative domains were deleted and superimposed on pre-defined declarative domains (Figs 1 and 2). The drawings were made in the *y*,*x* system, commonly used in shipping, where the axis *x* is typically adopted as the ship’s centre line.

**Table 3:** Parameters of medium ship absolute and relative effective domains after approximation.

Absolute domains		*x_0_*	*y_0_*	*a*	*b*
		[m]	[m]	[m]	[m]
	mean	28	15	1341	534
	max	125	34	1718	829
	min	-57	-7	537	439
Relative domains		*x_0_*	*y_0_*	*a*	*b*
		[L]	[L]	[L]	[L]
	mean	0.2	0.1	7.7	3.1
	max	0.7	0.2	9.9	4.8
	min	-0.3	0.0	3.1	2.5

L–length of the ship.

The real data presented in Figs 1 and 2 (dashed lines) together with approximated data (firm lines) confirm the validity of choosing the ellipse as the ultimate figure in approximating declarative domains. The relatively simple form of an ellipse equation enables further unambiguous analyses and, comparisons with elliptical effective domains.

The obtained declarative relative domains have a similar shape to the corresponding absolute domains, and the relative scale used makes them more readable for navigators. The above process was repeated for domains in three speed variants. In this case as well the obtained relative domains have a shape similar to the corresponding absolute domains, and the scale used gives additional possibility of comparison with other sources. The work [18] examines the relationship between domain size and shape and ship size and speed. This allows determining the declarative domain for ships of various sizes and various speeds.

### Effective domain

The simulation tests were conducted to identify effective domains of ships in an Electronic Chart Display and Information Systems (ECDIS) laboratory. In simulation tests, similarly to questionnaire surveys, only experienced deck officers and captains took part. In the tests, ship positions were recorded at the highest maximum frequency. The problem of determining the effective domain is described in detail in [10]. Example results—parameters of effective absolute and relative domains are shown in Table 3. The presented values are the coefficients of the parametric ellipse equation (here: elliptical domain):

- *x*_*0*_ –displacement of the centre along the axis x;
- *y*_*0*_ –displacement of the ellipse centre along the axis y;
- *a—*length of the semi-major axis;
- *b*–length of the semi-minor axis.

**Fig 3:**
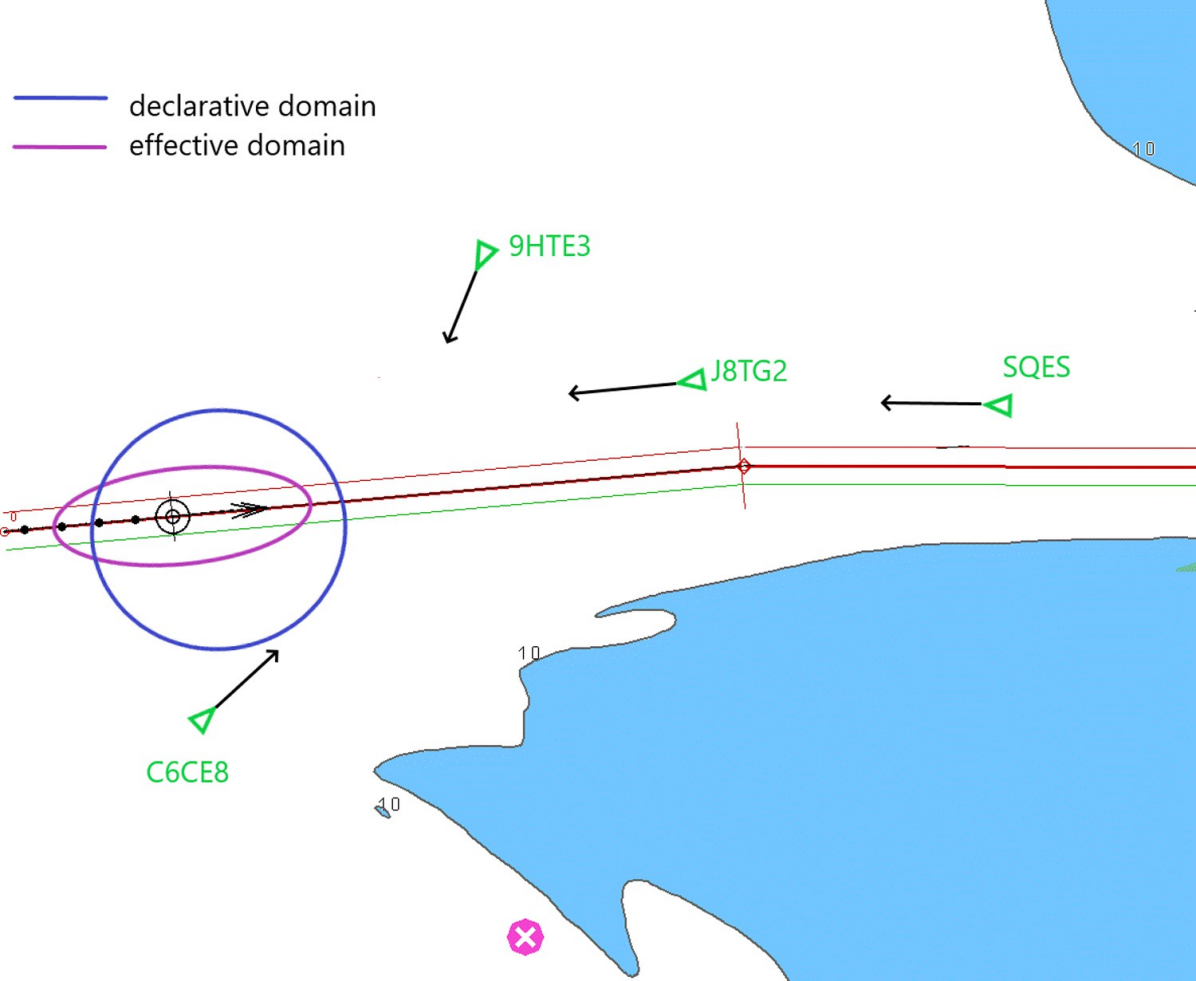
Absolute effective domains of a medium ship.

**Fig 4:**
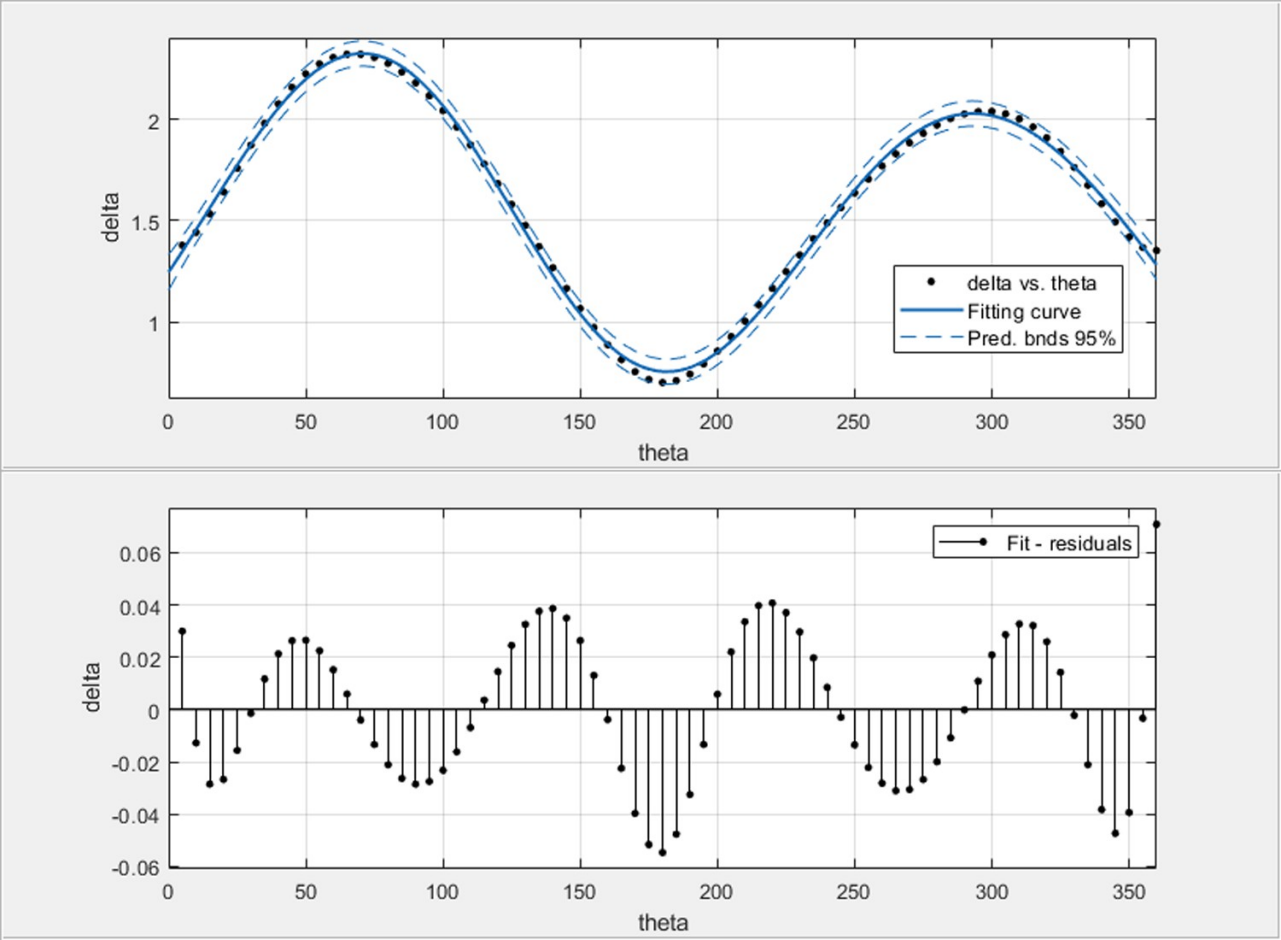
Relative effective domains of a medium ship.

Figs 3 and 4 depict selected effective domains plotted from the data in Table 3 (firm lines). The original data before approximation are also visible (dashed lines). The drawings were also made in the *y*, *x* system, commonly used in shipping.

The authors in [10] examine the relationship between domain size and shape and ship size and speed. This enables determining the effective domain for ships of various sizes and various speeds.

### The method for finding relationships between declarative and effective domains

**Fig 5:**
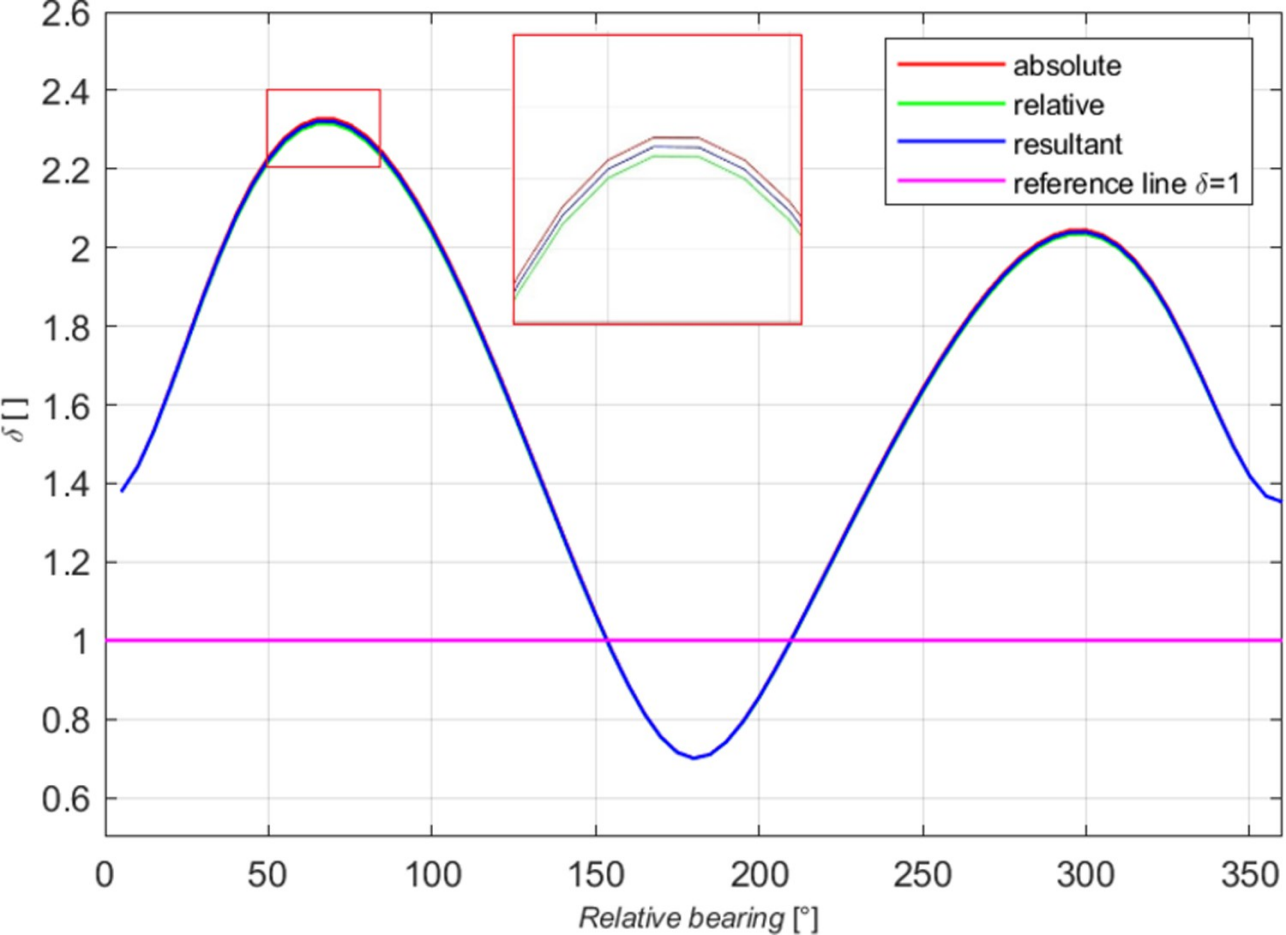
Selected corresponding declarative and effective ship domains.

The absolute declarative and effective domains of a medium ship are shown in Fig 5.

In a preliminary analysis of relationships between declarative and effective domains, where both domains were drawn on one chart, for all the sizes and speeds, it was found that identifying a simple function relationship would not be easy. The difficulty arises due to the shape, proportion, and displacement of ellipses’ centres of each domain. Besides, on some relative bearings the declarative distances are not greater than the effective ones, as could have been assumed after a superficial analysis.

**Fig 6:**
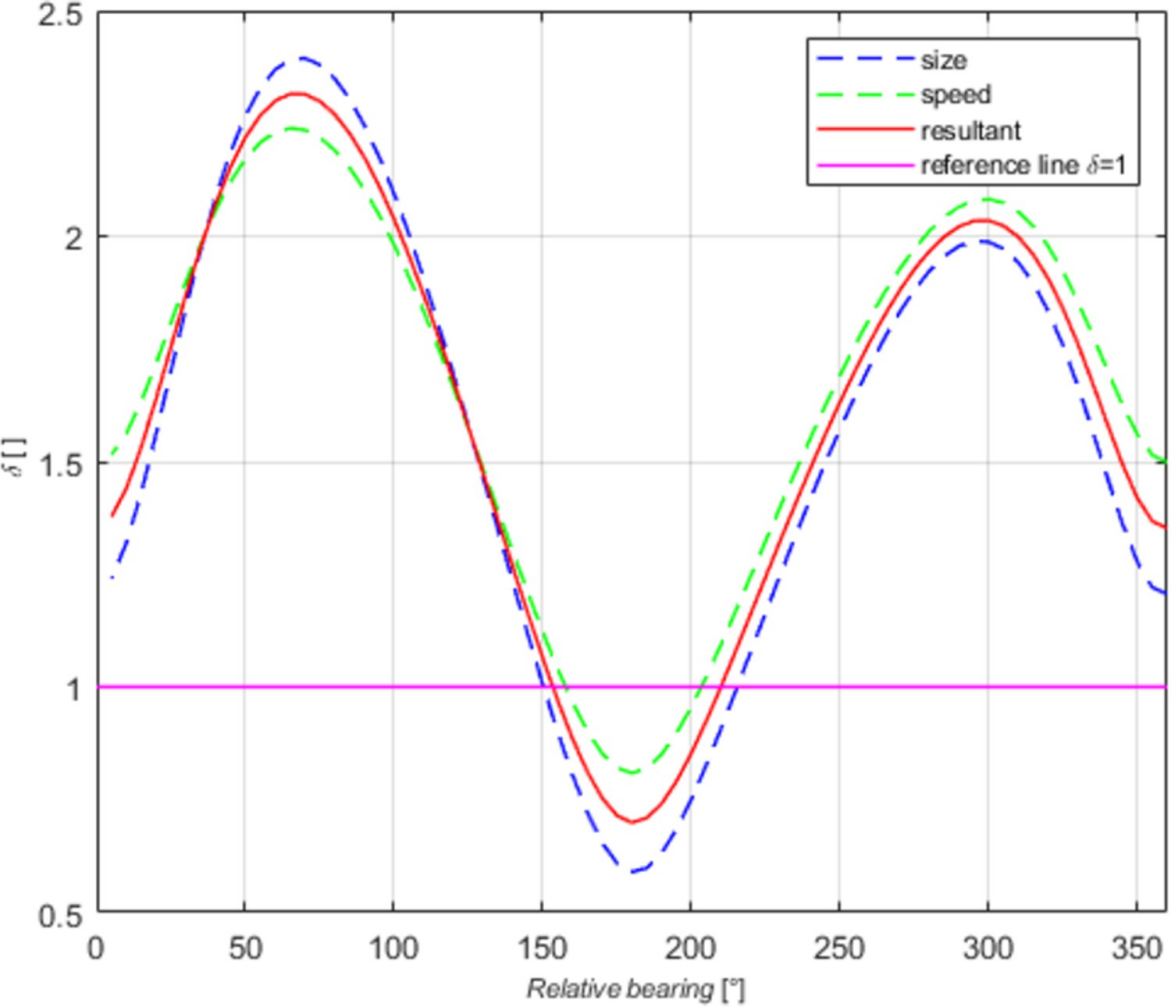
Identification of the relationship between the declarative domain and the effective domain.

The algorithm of determining relationships presenting a detailed description of the adopted method is shown in Fig 6.

**Fig 7:**
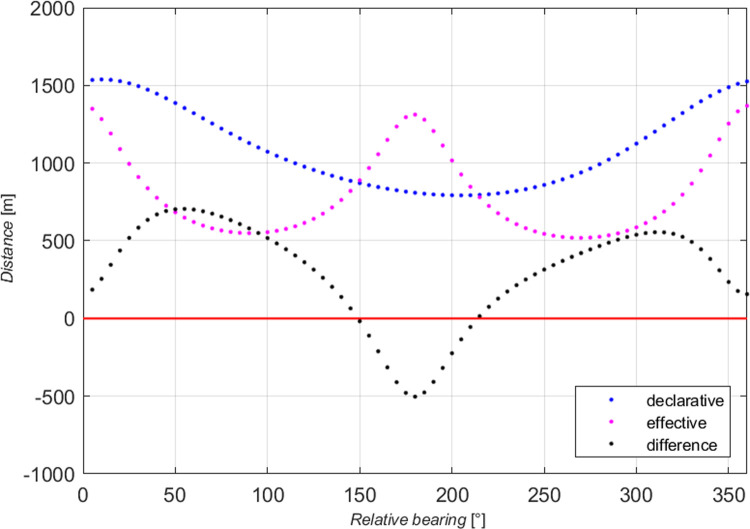
Determination of relationships between points of declarative and effective domains.

An auxiliary diagram for the analysis of relationships between domains is shown in Fig 7. We examined the distances on particular relative bearings measured from point S, the centre of the coordinate system (navigator’s position), to points E and D, intersections of the line defining a relative bearing (here: 045°) with, respectively, effective, and declarative domains. Described below is the method of determining relationships between the lengths of sections *d*_*D*_ and *d*_*E*_ on relative bearings, for *i*∈<000°; 360°>.

The relevant analysis requires preliminary verification of the consistency of the data held. Some doubts concerned the ambiguous interpretation of the relative bearing used for the comparison of distances. We checked whether the processing of data in the present form would not be subject to ambiguity and would not lead to incorrect conclusions.

The ellipses of all domains were written in the database as four previously indicated parameters (used in the ellipse equation): *x*_*0*_, *y*_*0*_, *a*, *b*. This enabled, after plotting the elliptical domains, to compare the distances *d*_*i*_ on each relative bearing *RB*_*i*_, that were direct equivalents of the radius *r* and angle θ applied to plot an ellipse. Each *RB*_*i*_ should be interpreted as relative for the domain under consideration because it is measured from its centre not overlapping the centre of another domain and the centre of the coordinate system. The direct comparison of such distances would lead to the derivation of only apparent relationship burdened with significant errors, depending on the type of the investigated domain. The coefficient defined and described further in this article (declarative distance to effective distance on *i-th* relative bearing) would take this form:

$$\delta_{i}^{\prime }=\frac{r_{iD}}{r_{iE}}$$ (1)

where

$\delta_{i}^{\prime }$—apparent coefficient *δ* on a relative bearing *i*;

*r*_*iD*_—apparent declarative passing distance on a relative bearing *i*;

*r*_*iE*_—apparent effective passing distance on a relative bearing *i* (Fig 7).

**Fig 8:**
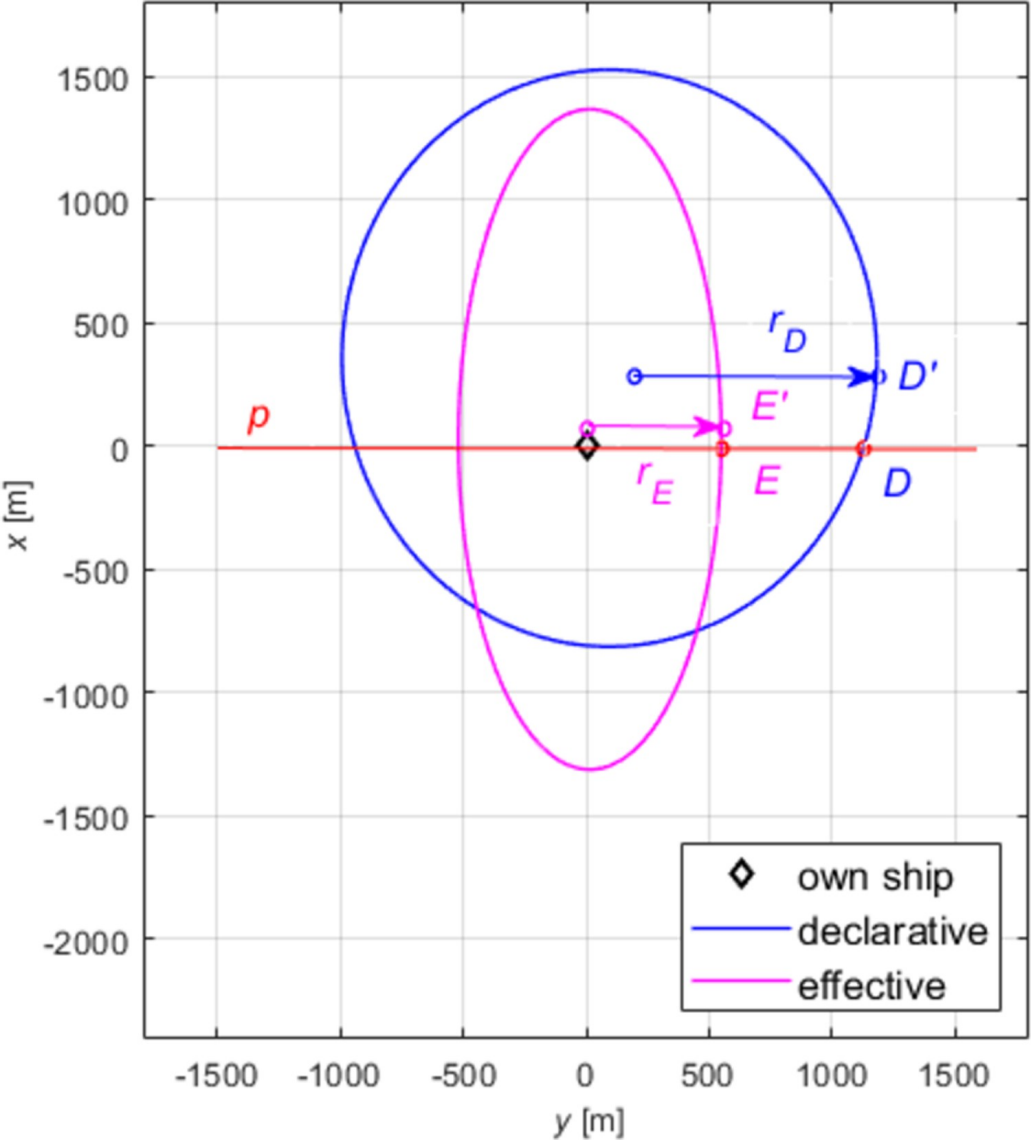
The impact of the position of the centre of the declarative domain and effective domain on the interpretation of the relative bearing. *r*_*D*_- radius of the declarative domain, declarative distance; *r*_*E*_- radius of the effective domain, effective distance; *p*—auxiliary straight line; *D*- point of the intersection of the ship’s beam with the declarative domain (RB = 090°); *D*′- point on RB = 090° taken from the centre of the declarative domain; *E*- point of the intersection of the ship’s beam with the effective domain (RB = 090°); *E*′- point on RB = 090° taken from the centre of the effective domain.

Due to the nature of the data (displaced ellipse centres relative to the coordinate system centre), to avoid ambiguities in Eq (3) that might arise due to differences in interpreting the relative bearing (visible in Fig 8), we calculated data to bring the ellipse centres to one point from which the relative bearing angles are determined.

The displacement of the domain ellipses’ centres seen in Fig 8 relative to the centre of the coordinate system leads to varied interpretation of the domain distance point on a selected relative bearing depending on the adopted centre (centre of the coordinate system, centre of the declarative domain ellipse or the centre of the effective domain ellipse). The red auxiliary straight line p indicates the ship’s port side and starboard side beam. If we analyse the starboard side, this is a line crossing both domains on the relative bearing *RB* = 090°. However, from the centre of the effective domain perspective, point E of the domain line crossing is visible on *RB*≅120°. From the centre of the declarative domain the point D of the domain line crossing the straight line pp is seen on *RB*≅095°, while the radiuses *r*_*E*_ and *r*_*D*_ indicate points E’ and D’ on apparent relative bearings 090°.

The coefficient determined below, describing the relationship of the declarative domain and the effective domain requires the unequivocal determination of declarative and effective distances on the same relative bearing with the navigator in the centre of the system. To identify the domain points on common relative bearings *RB*_i_, we solved the system of three Eq (4), of which the first two are equations of the elliptical declarative and effective domains, respectively, and the third one is the equation of straight line with the directional coefficient *e*. This straight line is passing through the centre of the coordinate system, crossing both ellipses, and determining the line of relative bearing *RB*_*i*_ on which the navigator sees the other ship.

$$\left\{ \begin{array}{l} \frac{{\left(x-x_{D0}\right)}^{2}}{a_{D}^{2}}+\frac{{\left(y-y_{D0}\right)}^{2}}{b_{D}^{2}}=1\\ \frac{{\left(x-x_{E0}\right)}^{2}}{a_{E}^{2}}+\frac{{\left(y-y_{E0}\right)}^{2}}{b_{E}^{2}}=1\\ y=e*x \end{array}\right.$$ (2)

where

*x*_*D*0_—displacement of the declarative domain centre on axis X;

*y*_*D*0_—displacement of the declarative domain centre on axis Y;

*a*_*D*_—semi-major axis of the declarative domain;

*b*_*D*_—semi-minor axis of the declarative domain;

*x*_*E*0_—displacement of the effective domain centre on axis X;

*y*_*E*0_—displacement of the effective domain centre on axis Y;

*a*_*E*_—semi-major axis of the effective domain;

*b*_*E*_—semi-minor axis of the effective domain;

*e*—directional coefficient of straight line—tangent (t).

As a result, we obtained the corresponding coordinates of the declarative ellipse points (*x*_*iD*_, *y*_*iD*_), and effective ellipse points (*x*_*E*_, y_E_) for the same relative bearing *RB*_*i*_ from the common centre of the system—ship’s position (points D, E)—not the geometric centre of one of the elliptical domains. Only such pairs of coordinates of the examined domain points permit unequivocal comparison of the distances determined by those points.

The most convenient and easily interpretable method for determining relationships is the presentation of the difference of the absolute distances between domain points *τ* (5) as the function of the relative bearing (see Fig 7).

$$\tau_{i}=d_{iD}-d_{iE}$$ (3)

where

*τ*_*i*_—difference between the declarative and effective distances on relative bearing *i*;

*d*_*iD*_—declarative passing distance on relative bearing *i*;

*d*_*iE*_—effective passing distance on relative bearing *i*.

**Fig 9:**
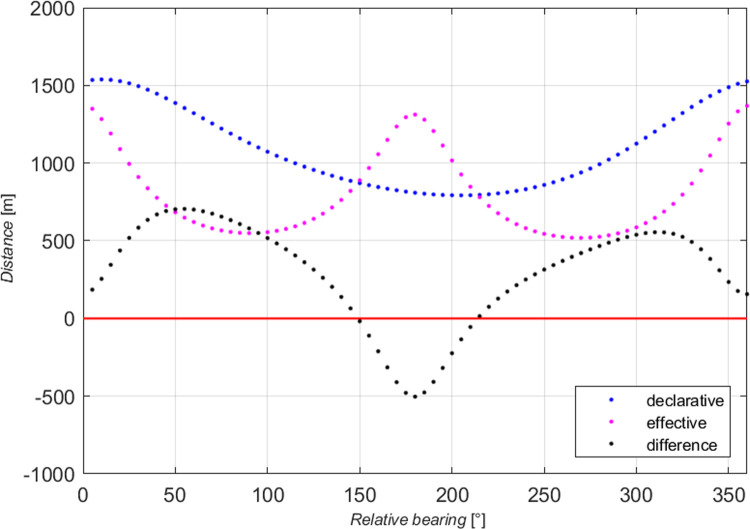
The difference of the distances *τ* as the function of the relative bearing for selected declarative and effective domains.

The relevant graphic summary is shown in Fig 9.

The blue colour marks the values of declarative distances, purple—effective distances, while the differences of distances on the given *RB* are depicted in black. The red line is the reference line for *τ* = 0. Due to doubts that may occur in interpreting negative values of the distance differences and difficulties in the presentation of values *τ* in the 360-degree system commonly used by navigators, the relationship between the domains is shown as the ratio of distances *d*_*iD*_ and *d*_*iE*_ (6).

$$\delta_{i}=\frac{d_{iD}}{d_{iE}}$$ (4)

where

*δ*_*i*_—coefficient *δ* on relative bearing *i*;

*d*_*iD*_—calculated declarative passing distance on relative bearing *i*;

*d*_*iE*_—calculated effective passing distance on relative bearing *i* (see Fig 7).

## Results

### The relationship between the declarative and effective domains

**Fig 10:**
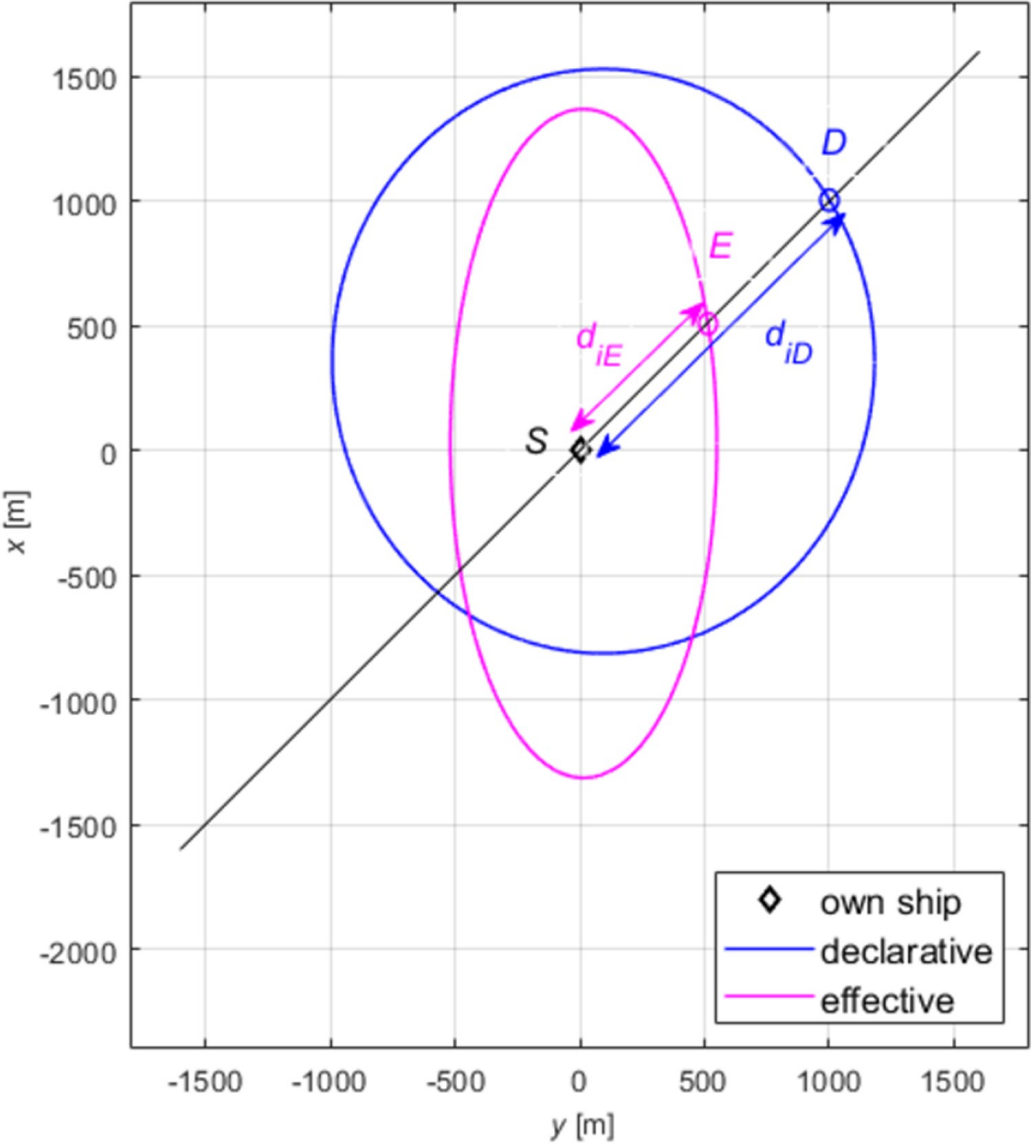
The coefficient δ_B_ as the function of the relative bearing for ships of different size and moving at various speeds.

Intermediate results were analysed in detail. The coefficients *δ* of the mean domains in all the cases examined were compared. The domain values were averaged and a function linking the declarative and the effective domains was formulated. The relationship between the coefficients δ_B_ (absolute domains) is shown in Fig 10, where the curves obtained after averaging the coefficients for ships of different size and moving at various speeds. The similarity of the curves allows us to generate a resultant curve (red). The purple colour represents a reference line of the coefficient δ_B_ = 1 for simpler interpretation of the chart (the values above 1 mean the declarative distance δ times larger than the effective distance, while for the values below 1 the declarative distance is shorter than the effective distance). Similar relationships were found for relative domains by analysing the coefficient δ_W_.

**Fig 11:**
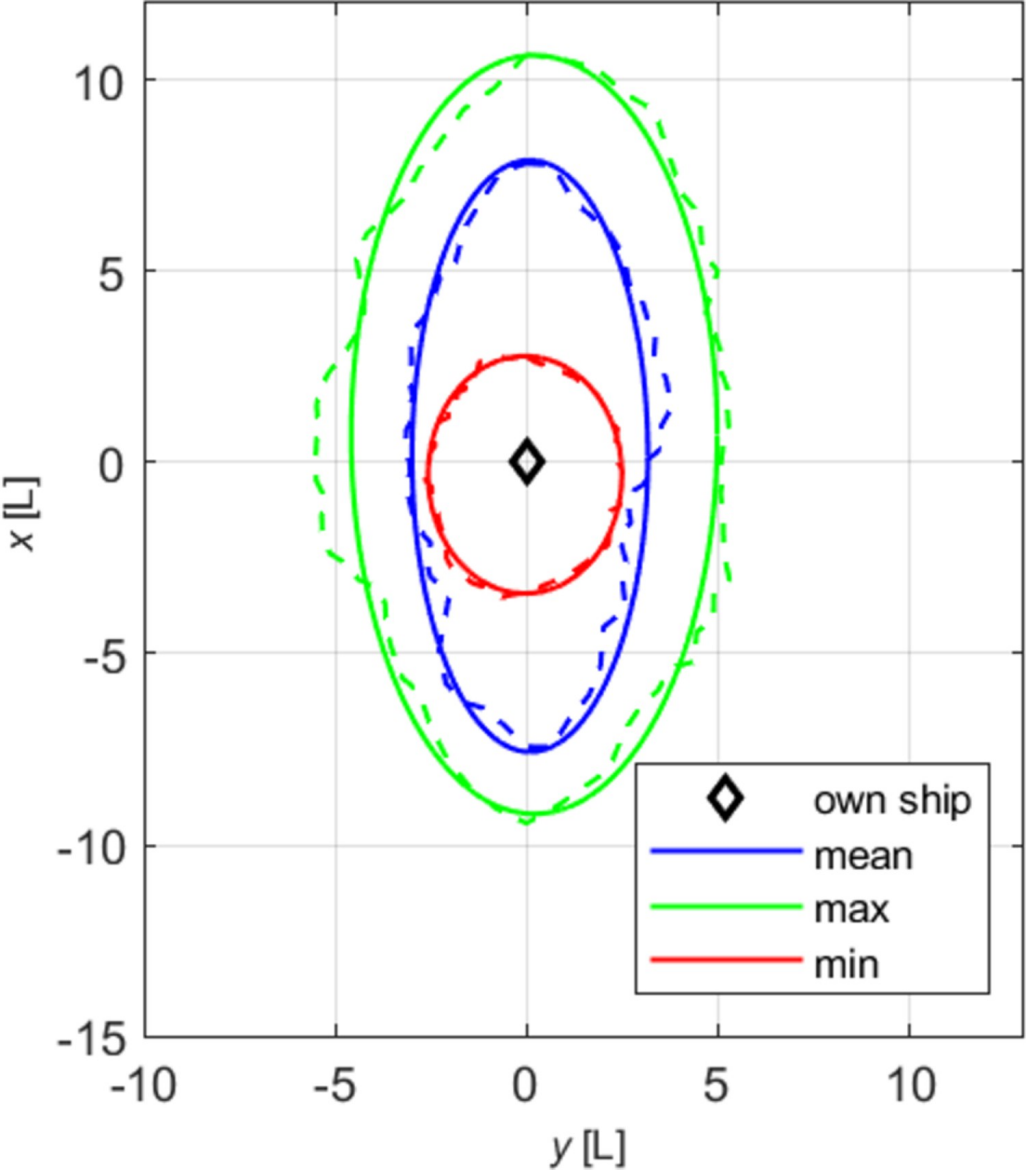
The resultant coefficients *δ*_*B*_, *δ*_*W*_ and averaged *δ* as the function of the relative bearing for all the tests.

Further in the analysis of changes in the coefficient δ values, the absolute coefficient δ_B_ and relative coefficient δ_W_ were plotted in one diagram for all types of ships and speed variants (Fig 11). Both curves are found to run similarly, and the differences between the coefficient values on particular relative bearings were below 0.35% of the values. The overlapping curves are shown in Fig 11, where red box indicates slight differences between the curves shapes. Individual curves are difficult to identify in the diagram because they practically overlap. The slight differences in the curves result from the method of approximation of domain points. Genetic algorithms and the same fitting function were used to separately approximate each absolute and relative domain. The differences, however, were in the lower and upper limits of the variables (*x*_*0*_, *y*_*0*_, *a*, *b*), individually identified for each domain. We found that this will be a more precise method than a simple division of subsequent absolute distances by the appropriate length of the ship.

The obtained curve (blue in Fig 11) demonstrates the overall relationship between the declarative domain and the effective domain of the ship. To display the curve as a function, it was approximated. Having analysed several applicable approximating functions, we decided to use the model of the periodic fitting function of the sum of sines, mainly due to the sinusoid nature of the curve.

The model function of the sum of sines is described by the general Eq (7):

$$y=\sum_{i=1}^{n}a_{i}sin\;(b_{i}x+c_{i})$$ (5)

where

*n*—number of terms of the equation in the series (1≤*n*≤8);

*a*_*i*_—amplitude constant;

*b*_*i*_—frequency constant;

*c*_*i*_—phase constant for each term (sinusoidal wave) of the equation.

The approximation function was analysed for various number of terms. The fitting was done in variants from n = 1 to n = 8 terms (sinusoidal waves). Due to the sufficient fit, the chosen function was *δ = f*_*(RB*),_ a three-term function of the sum of sines (8).


$$y=a_{1}\;\sin\left(b_{1}x+c_{1}\right)+a_{2}\;\sin\left(b_{2}x+c_{2}\right)+a_{3}\;\sin\left(b_{3}x+c_{3}\right)$$ (6)

Then the function dependence of the coefficient *δ* on the relative bearing *RB* assumes the form (9):

$$\delta (RB)=a_{1}\;\sin\left(b_{1}RB+c_{1}\right)+a_{2}\;\sin\left(b_{2}RB+c_{2}\right)+a_{3}\;\sin\left(b_{3}RB+c_{3}\right)$$ (7)

where

*RB*—relative bearing;

*a*_1_, *b*_1_, *c*_1_, *a*_2_, *b*_2_, *c*_2_ *a*_3_, *b*_3_, *c*_3_—function coefficients (Table 4).

**Table 4:** The equation coefficients δ as the function of relative bearing.

coefficient	*a*_*1*_	*b*_*1*_	*c*_*1*_	*a*_*2*_	*b*_*2*_	*c*_*2*_	*a*_*3*_	*b*_*3*_	*c*_*3*_
value	1.570	0.003	1.106	0.724	0.026	-0.082	0.101	0.039	-1.753
lower limit	1.424	-0.001	0.227	0.607	0.022	-0.766	0.026	0.035	-2.615
upper limit	1.716	0.007	1.985	0.841	0.030	0.603	0.176	0.044	-0.890

The function coefficients with their lower and upper limits at the confidence level of 95% are shown in Table 4. The goodness of fit can be improved by increasing the number of the terms of the equation.

For the equation thus written, the goodness of fit is determined by the following parameters:

- sum of squared estimate of errors, SSE: 0.05476;
- coefficient of determination, R-square: 0.9967;
- adjusted R-square: 0.9962;
- root mean square error, RMSE: 0.02948.

**Fig 12:**
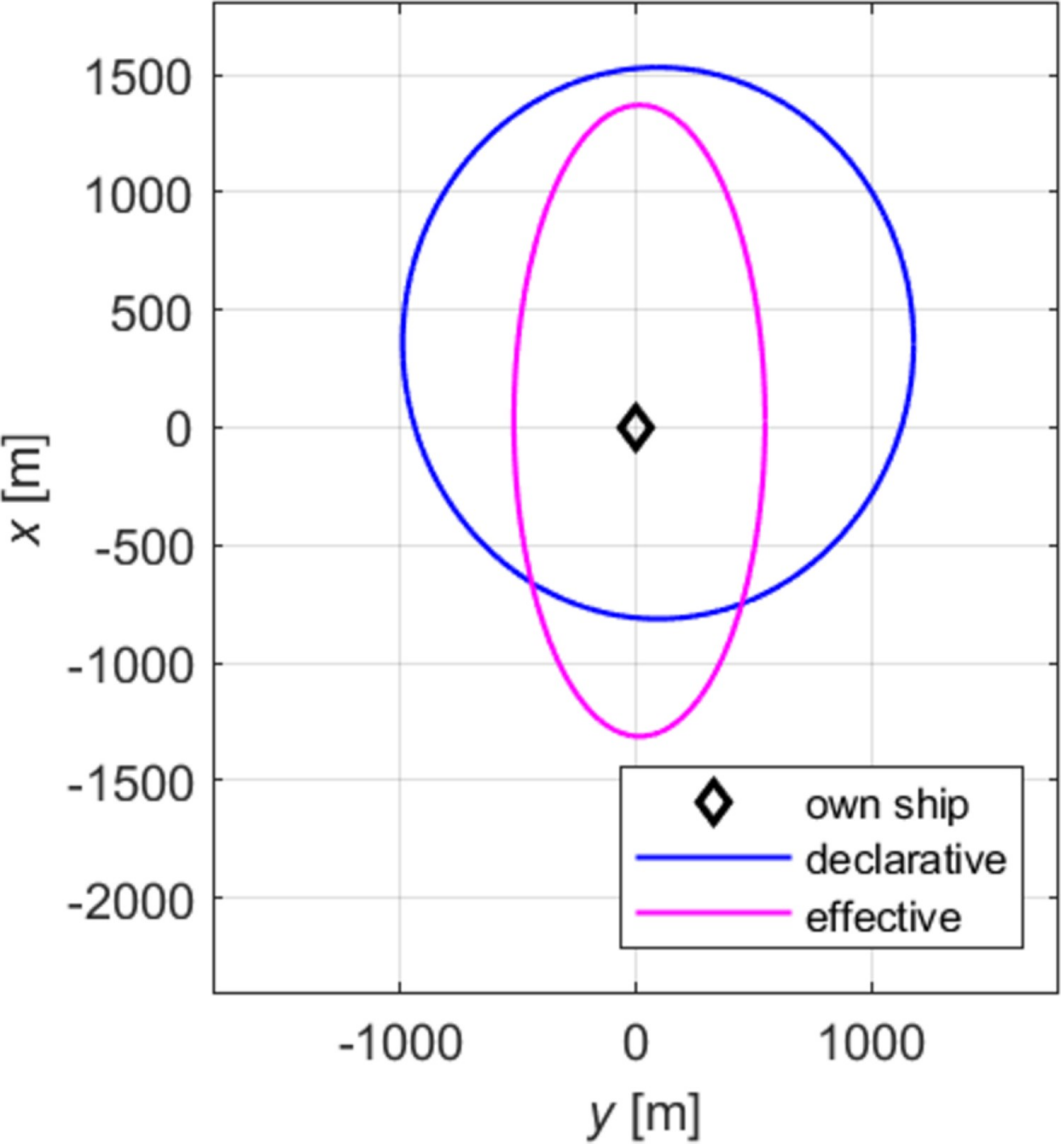
The result of the approximation of the coefficient δ curve with the plot of the prediction error values.

The result of approximation is shown in Fig 12. It contains the values of coefficient δ as the function of relative bearing discretely changed every 5 degrees, the curve of approximation and prediction boundaries at 95% confidence level and an additional chart of prediction error values.

### An example of the method application

With knowledge of the relationship between the declarative and effective domains, it is possible to determine the actual passing distance between two ships, based on the assumed and desired value. This takes into account differences in the domains caused mainly by manoeuvring characteristics of ships, as well as navigators’ habits and practices.

**Fig 13:**
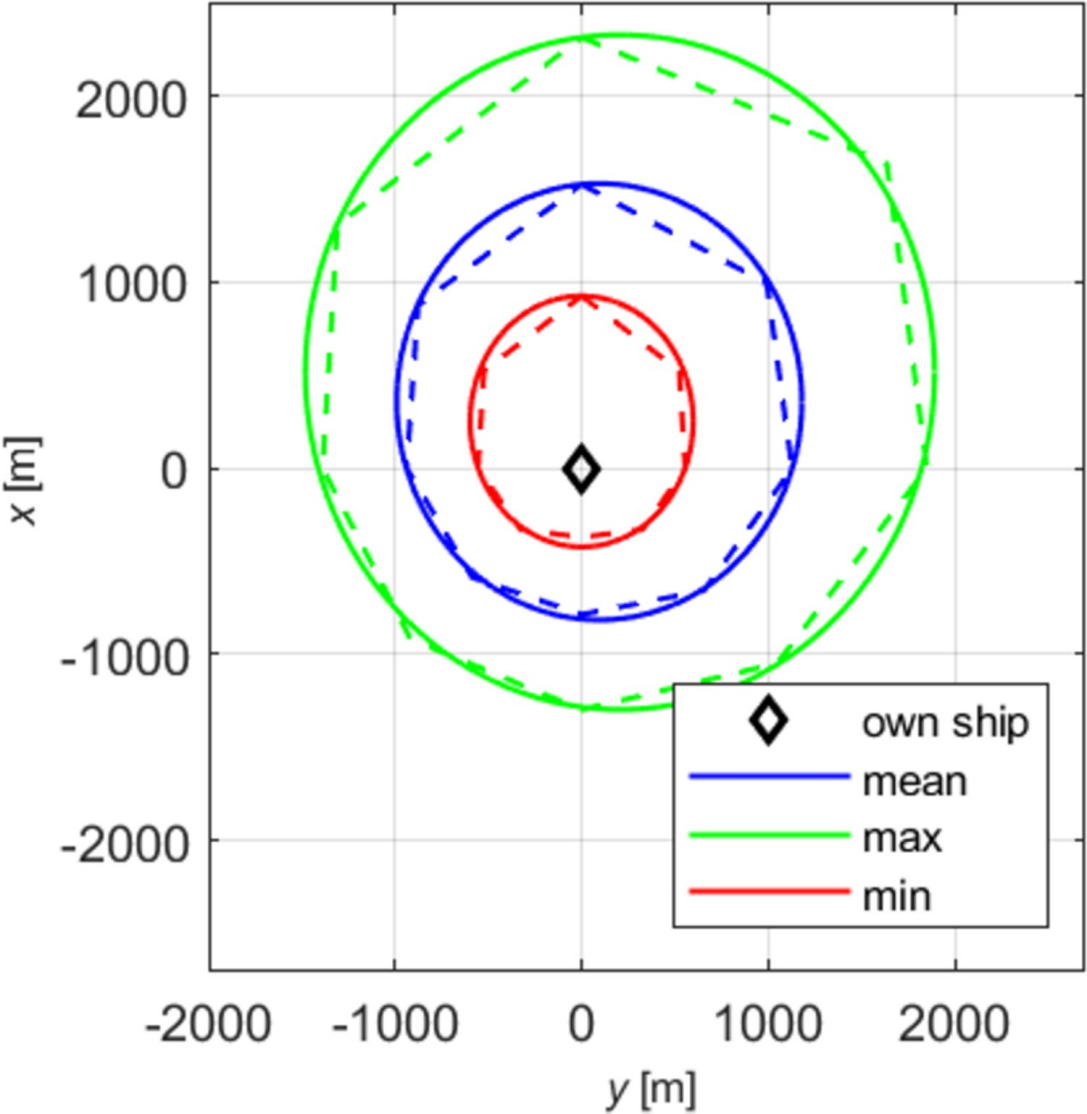
The proposed declarative and effective domains in the ECDIS system.

An example of practical use of the established relationship is shown in Fig 13. It depicts a navigational situation in the ECDIS system with an AIS overlay. Target ships are presented as AIS objects: green triangles with call signs and movement vectors. Own ship is marked as a standard symbol: a black circle with the movement vector and the trace. Own ship’s track is currently monitored (firm red line) with cross-track deviation limits established at the planning stage (thin solid green and red lines that can be crossed if necessary, e.g. for manoeuvres). In addition, the domains are shown now: previously identified declarative domain (blue) and the effective domain (purple) calculated from the declarative domain. An analysis using collision avoidance functions of the ECDIS or ARPA systems allows us to assess that the ships with call signs J8TG2 and SQES will cross the declarative domain of own ship, while staying outside the effective domain. Ships 9HTE3 and C6CE8 will violate the effective domain.

**Fig 14:**
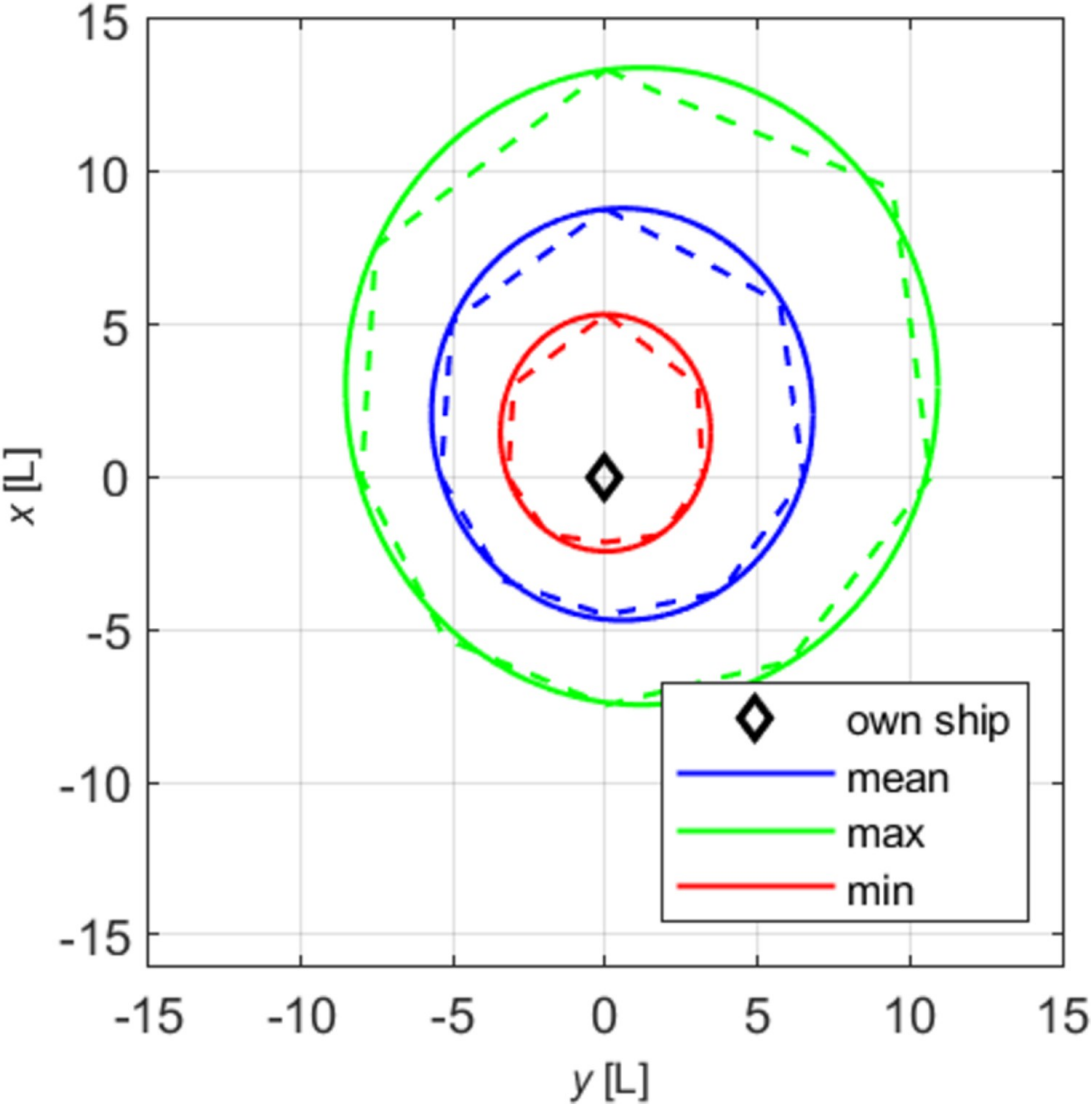
A navigational situation after six minutes.

The violation of the declarative domain in the situation herein considered is not desirable, but acceptable and justified by area restrictions, while the violation of the effective domain will lead to a close quarters situation. If the ships HTE3, C6CE8 or own ship fail to commence manoeuvres, the effective domain of own ship will be violated. The navigational situation after six minutes is presented in Fig 14. Ships J8TG2 and SQES will only violate the declarative domain. The declarative and effective domains depicted in the ECDIS system will allow the navigator to detect early enough a case of effective domain violation (Fig 13), pushing him to take timely, COLREG-compliant action to avoid a collision.

## Conclusions

The authors present methods of determining declarative and effective domains. Their analysis encompasses ship encounters in restricted waters. The declarative and effective domains are displayed as ellipses that vary in size, lengths of ellipse semi-axes and the displacement of the centre. The comparison of declarative domains with effective domains was made to find and define a relationship between them. The relationship between the declarative domain and effective domain allows predicting passing distance based on the declared distance of passing the target, thus determining ship’s behaviour in an encounter with another ship. Based on this basis, the navigational risk of the encounter is estimated and, if necessary–action reducing the risk.

The formulated relationship allows the navigator to determine an effective passing distance based on the passing distance he assumes. An example of practical use of the established relationship is given. It may be worth considering the use of the declarative domain identified on the basis of the determined effective domain, e.g., to assess the navigator’s profile. The identification of the declarative and effective domains is important from the viewpoint of monitoring and assessment of the navigational situation of a proceeding ship, thus increasing the situational awareness of the navigator. The ship domain can be used as a safety criterion in the process of determining the safe trajectory of ship movement in a collision situation.

The authors’ research is aimed at methods of determining ship domains of traditionally manned ships. However, the authors believe that the proposed methods and the domain determined on their basis will find applications on autonomous ships of various degrees of autonomy. Given the fact that the process of introducing autonomous ships will be gradual and extended in time, ships’ encounters in the years to come will involve the whole spectrum of ships: fully manned vessels, remotely controlled vessels with skeleton crews or unmanned, or fully autonomous ships. In each variant of encounter, the knowledge of navigational safety assessment criteria used by these ships will allow determining or negotiating safe ship movement trajectories, contributing to the safety of navigation and collision prevention.

## Supporting information

S1 Appendix Questionnaire no. 1. (PDF)

S2 Appendix Questionnaire no. 2. (PDF)

S3 Appendix Questionnaire no. 3. (PDF)
