# Supplementary material for: The ship domain in navigational safety assessment
Source: PLoS One. 2022 Apr 26;17(4):e0265681. doi: 10.1371/journal.pone.0265681 (PMC9041838; doi:10.1371/journal.pone.0265681)
Supplement: S1 Appendix — (PDF) [file pone.0265681.s001.pdf]

Questionnaire no. 1

You are on a vessel with the given parameters in the restricted area - Singapore Strait (see displayed sea chart).

Enter the distance (Nautical Miles) you wish to be passed by **<u>the similar size vessel</u>**, proceeding at a similar speed - depending on the relative bearing at which it was sighted.

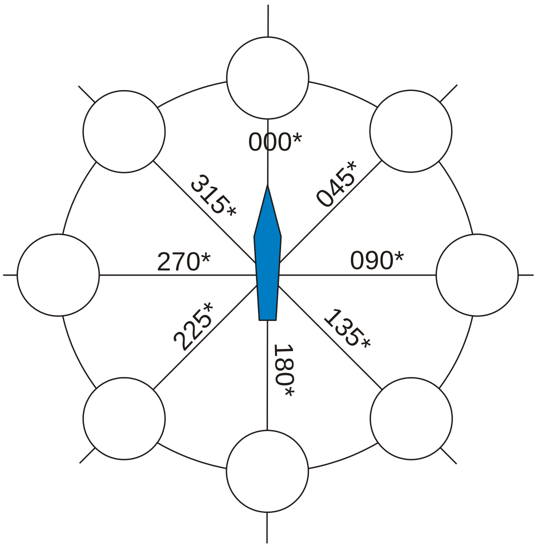

Large ship:

Deadweight (DWT): 63 430 t

Length over all (LOA): 261.0 m

Breadth (B): 48.0 m

Draught (T): 9.0 m

Speed (v): 16.3 knots

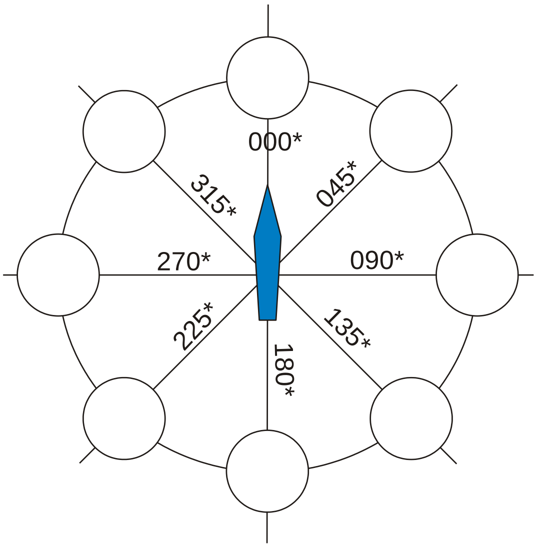

Medium ship

DWT: 19 512 t

LOA: 174.0 m

B: 23.0 m

T: 8.1 m

v: 18.9 knots

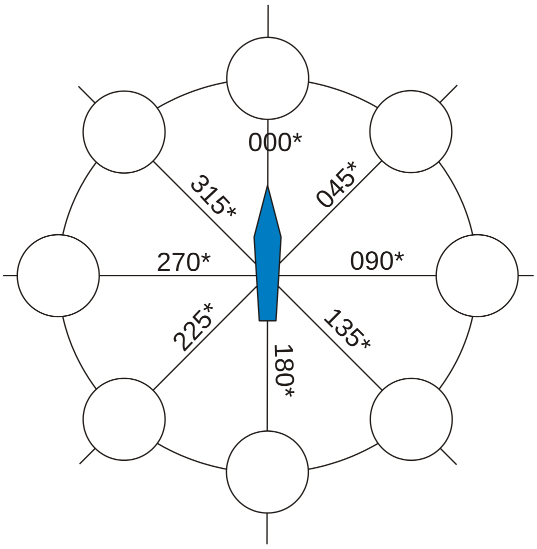

Small ship

DWT: 3 510 t

LOA: 95.0 m

B: 13.0 m

T: 3.7 m

v: 11.1 knots
