# Supplementary material for: The ship domain in navigational safety assessment
Source: PLoS One. 2022 Apr 26;17(4):e0265681. doi: 10.1371/journal.pone.0265681 (PMC9041838; doi:10.1371/journal.pone.0265681)
Supplement: S3 Appendix — (PDF) [file pone.0265681.s003.pdf]

Questionnaire no. 3

You are on a vessel with the given parameters in the restricted area - Dover Strait (see displayed see chart).

Enter the distance (Nautical Miles) you wish to be passed by **the same size vessel**, proceeding at **twice the speed** - depending on the relative bearing at which it was sighted.

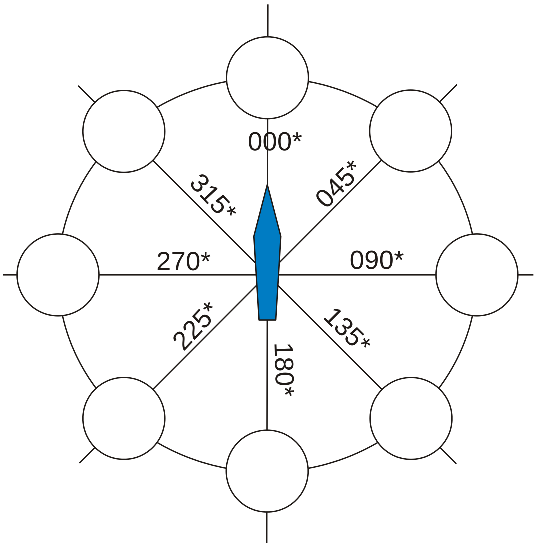

Large ship:

Deadweight (DWT): 63 430 t

Length over all (LOA): 261.0 m

Breadth (B): 48.0 m

Draught (T): 9.0 m

Own ship peed (v): 8,1 knots

Target ship speed (v_o): 16.3 knots

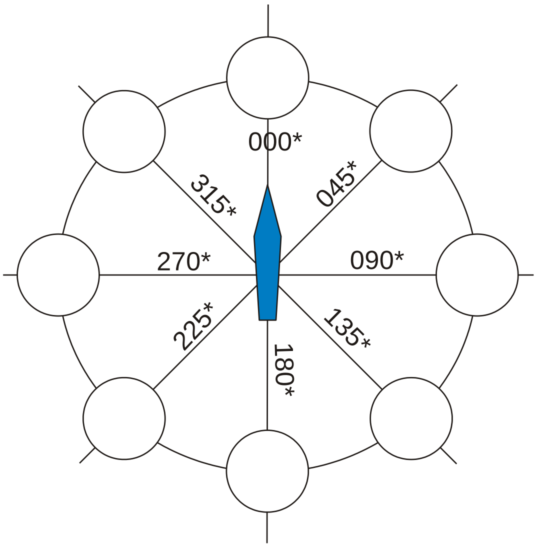

Medium ship

DWT: 19 512 t

LOA: 174.0 m

B: 23.0 m

T: 8.1 m

Own ship speed (v): 10.0 knots

Target ship speed (v_o): 18.9 knots

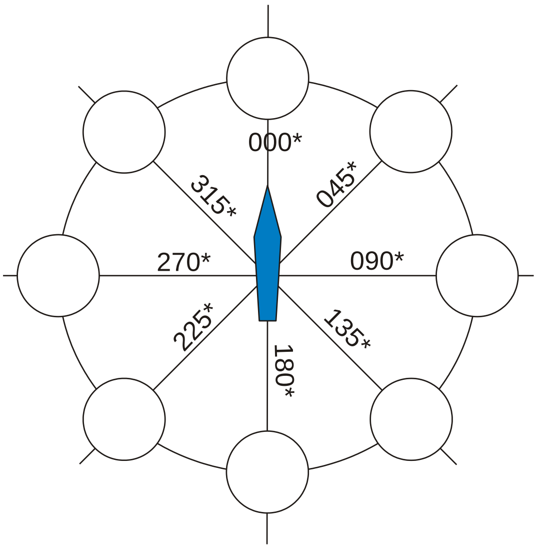

Small ship

DWT: 3 510 t

LOA: 95.0 m

B: 13.0 m

T: 3.7 m

Own ship speed (v): 5.6 knots

Target ship speed (v_o): 11.1 knots
